# Tertiary lymphoid structures in colorectal cancer

**DOI:** 10.1080/07853890.2024.2400314

**Published:** 2024-11-22

**Authors:** Jianyu Lv, Xiuyu Zhang, Mi Zhou, Junbin Yan, Guanqun Chao, Shuo Zhang

**Affiliations:** a Department of Gastroenterology, The Second Affiliated Hospital, Zhejiang Chinese Medical University, Zhejiang, China; b Department of General Practice, Sir Run Run Shaw Hospital, Zhejiang University, China

**Keywords:** Tertiary lymphoid structure, colorectal cancer, microenvironment, immunotherapy, prediction

## Abstract

**Background:**

Tertiary lymphoid structures (TLS) are ectopic clusters of immune cells found in non-lymphoid tissues, particularly within the tumor microenvironment (TME). These structures resemble secondary lymphoid organs and have been identified in various solid tumors, including colorectal cancer (CRC), where they are associated with favorable prognosis. The role of TLS in modulating the immune response within the TME and their impact on cancer prognosis has garnered increasing attention in recent years.

**Objective:**

This review aims to summarize the current understanding of TLS in CRC, focusing on their formation, function, and potential as prognostic markers and therapeutic targets. We explore the mechanisms by which TLS influence the immune response within the TME and their correlation with clinical outcomes in CRC patients.

**Methods:**

We conducted a comprehensive review of recent studies that investigated the presence and role of TLS in CRC. The review includes data from histopathological analyses, immunohistochemical studies, and clinical trials, examining the association between TLS density, composition, and CRC prognosis. Additionally, we explored emerging therapeutic strategies targeting TLS formation and function within the TME.

**Results:**

The presence of TLS in CRC is generally associated with an improved prognosis, particularly in early-stage disease. TLS formation is driven by chronic inflammation and is characterized by the organization of B and T cell zones, high endothelial venules (HEVs), and follicular dendritic cells (FDCs). The density and maturity of TLS are linked to better patient outcomes, including reduced recurrence rates and increased survival. Furthermore, the interplay between TLS and immune checkpoint inhibitors (ICIs) suggests potential therapeutic implications for enhancing anti-tumor immunity in CRC.

**Conclusions:**

TLS represent a significant prognostic marker in CRC, with their presence correlating with favorable clinical outcomes. Ongoing research is required to fully understand the mechanisms by which TLS modulate the immune response within the TME and to develop effective therapies that harness their potential. The integration of TLS-focused strategies in CRC treatment could lead to improved patient management and outcomes.

## 1. Introduction

Lymphoid organs, such as the thymus, spleen, and lymph nodes (LN), are a core component of the mammalian immune system [1]. They are divided into primary lymphoid organs (thymus and bone marrow) and SLOs (such as the spleen and LN), which are responsible for generating lymphocytes from immature progenitor cells and coordinating immune responses, organizing interactions of effector immune cells in space [2]. Tertiary lymphoid structures (TLS), referred to as tertiary lymphoid organs or ectopic lymphoid structures, are clusters of immune cells found in non-lymphoid tissues. These structures exhibit both structural and functional characteristics that closely resemble those of SLOs like LN. This includes the presence of the T cell zone containing mature dendritic cells (DC), the germinal center populated by follicular dendritic cells (FDC), as well as proliferating B cells and high endothelial venules (HEV) [3]. TLS is produced in an immune-driven inflammatory environment, such as infection, transplant rejection, autoimmune diseases, and tumors, as a response to immune-driven inflammation [4].

Tumors develop in a complex tumor microenvironment (TME). Cells surrounding or within the tumor, such as stromal cells, endothelial cells, innate cells, and lymphocytes, can interact with tumor cells to regulate tumor growth, invasion, and metastasis [5,6]. A successful immune response against tumors necessitates the activation, presence, and co-stimulation of all elements in the lymphatic system. The identification of TLS inside tumors validates this idea, establishing a nearby immune microenvironment that stimulates cellular and humoral immune responses against tumors, thus initiating enhanced immune responses [7]. TLS has been observed in different types of tumors, such as hepatocellular carcinoma [8], lung cancer [9], colorectal cancer (CRC) [10], and breast cancer [11].

In 2020, there were more than 1.88 million new cases of CRC, a prevalent malignant tumor in the digestive tract, resulting in 910,000 deaths, as reported in global cancer statistics [12]. CRC is currently one of the main malignant tumors. Global cancer data for 2020 shows that the incidence rate of CRC is about 10% (10393/100,000), ranking third in the world’s cancer incidence rate, and the mortality rate is about 9.2% (9256/100,000), ranking second in the world’s cancer mortality rate [12]. The lack of reliable prognostic markers and treatment strategies has always been a bottleneck in the diagnosis and treatment of CRC. Increasing evidence supports the importance of the immune microenvironment in cancer development [13]. The TME and immune monitoring status represent key prognostic markers for CRC. Manipulation of TLS neogenesis and maintenance may lead to persistent adaptive anti-tumor responses, which may represent an effective treatment strategy for CRC.

## 2. CRC and TILs

The TME consists of a mix of cancer cells, stromal cells, immune/inflammatory cells, extracellular matrix, blood vessels, secreted proteins, signaling molecules, and RNA [14–19]. This complex mixture plays a crucial role in tumor progression and treatment resistance. The complex components of TME interact with each other in a non-autonomous manner, affecting tumor biology and immune therapeutic responses, regulating tumor cell proliferation, cell death, angiogenesis, energy metabolism, immune evasion, promoting inflammation, invasion, and metastasis, all of which contribute to tumor progression [20–23]. In CRC, the communication between cells in the TME plays a crucial role in the progression of the disease. Inflammation plays a key role in driving the plasticity of tumor cells and surrounding cells within the TME, impacting different stages of tumor development. This interaction between cells and inflammation in the TME is a significant factor in the aggressiveness of CRC [24]. The inflammatory environment is of significant importance throughout all the stages of CRC. TME is a local site of inflammation, and epithelial tumor tissue continuously interacts with TME cells through cytokines, chemokines, and growth factors. The inflammatory environment plays a dual role in tumor development. On the one hand, it contributes to tumorigenesis by producing reactive oxygen species and inducing epigenetic changes. On the other hand, it promotes tumor growth by providing growth factors and pro-inflammatory cytokines.

Infiltrating immune cells within tumors, known as tumor-infiltrating lymphocytes (TILs), display a diverse range across patients. This cellular mix encompasses various types such as T lymphocytes, B lymphocytes, natural killer (NK) cells, macrophages, and additional components of the innate immune system. Variations in the quantity, classification, and functionality of TILs can have a significant impact on a tumor’s vulnerability to immune-mediated countermeasures [25]. Pre­dominantly, TILs consist of T and B lymphocytes along with NK cells [26]. Among these, T and B lymphocytes constitute the bulk of the immune cell population found within TLS. Therefore, the following discussion will focus on detailing the roles of these two predominant TIL subsets within the colorectal TME.

### 2.1. T Cells

A substantial body of evidence indicates that T cells play a pivotal role in anti-tumor immune responses. T cells represent the most prevalent immune cells in CRC and are subdivided into CD8+ T cells and CD4+ T cells. The majority of T cells possess T cell receptors comprising two chains: α chain and β chain [27]. In the context of the CRC TME, TILs represent a heterogeneous mixture of adaptive immune cells, comprising mainly anti-tumour T cells (CD4+ and CD8+ subsets) and suppressive CD4+ T cells (Treg) [28]. T cells are responsible for the prevention of tumour growth by attacking cancer cells. CD8+ T cells, also known as cytotoxic T lymphocytes (CTLs), recognize antigens presented by MHC class I molecules on the surface of APCs, usually DCs. CTLs are instrumental in the destruction of cancerous cells, predominantly utilizing the Fas-FasL and perforin-granzyme mechanisms, complemented by the action of cytokines interleukin-2 (IL-2), interleukin-12 (IL-12), and interferon-gamma (IFNG) [29]. A heightened presence of CTLs within the tumor milieu correlates positively with counteracting tumor growth and is indicative of enhanced patient outcomes across a spectrum of cancer types [30]. Conversely, as the severity of the tumor escalates, the density of CTLs tends to diminish [31]. In CRC, the spectrum of CD4+ T cells include Th1, Th2, Th17, Th22, regulatory T cells, and T follicular helper (Tfh) cells [32]. Th1 cells are pivotal in the secretion of IL-2, TNF, and IFNG, all of which are integral to the immune response against tumors [33]. The activation of CD8+ T cells by Th1 cells is a key mechanism through which they exert their anti-tumor influence [34]. Th2 cells, in contrast, are characterized by their secretion of interleukin-4 (IL-4), interleukin-5 (IL-5), and interleukin-13 (IL-13), which can contribute to the transition from colitis to cancer [35]. Th17 cells are implicated in fostering tumor growth in CRC and other malignancies [36]. The cytokines interleukin-17 (IL-17) and interleukin-22 (IL-22), key players in Th17 cell function, with IL-17A, a variant of IL-17, being particularly implicated in the advancement and angiogenesis of CRC through the signal transducer and activator of transcription 6 (STAT6) signaling pathway [37]. In human CRC, Th22 cell infiltration is associated with favorable prognosis [38]. The role of Treg cells in CRC remains controversial, and analysis of TILs in human CRC has shown that two heterogeneous subpopulations of forkhead box P3 (FOXP3) + T cells are associated with patient prognosis [39]. CRCs with abundant FOXP3(lo) T cell infiltration show significantly better prognosis than those with mainly FOXP3(hi) Treg cell infiltration, producing a strong pro-inflammatory environment by secreting inflammatory cytokines such as transforming growth factor-beta (TGF-β), IL-12, and TNF [39]. A large number of Tfh cells in CRC is associated with good prognosis [40]. Tfh cells promote B cell activation and antibody conversion into anti-tumor IgG1 and IgG3 through IL-21, thereby promoting B cell-mediated humoral anti-tumor immunity [41] (Table 1).

**Table 1.** The cytokines secreted by T cell subpopulations and their impact on tumor production.

T cell subpopulation	Cytokines	Impact on tumors	Reference
Th1	IL-2, TNF, IFNG	Inhibit	Schmitt et al. 2015 [33]
Th2	IL-4, IL-5, IL-13	Inhibit	Braumüller et al. 2022 [35]
Th17	IL-17, IL-22, IL-3, IL-68A	Promote	Robert et al. 2011 [36]
Treg	TGF-β, IL-12, TNF	Promote	Saito et al. 2016 [39]
Tfh	IL-21	Inhibit	Niogret et al. 2021 [41]
CTL	IL-2, IL-12, IFNG	Inhibit	Hiam-Galvez et al. 2021 [29]

### 2.2. B Cells

B lymphocytes play a pivotal role in the adaptive immune system, contributing to various immune functions and primarily acting as effectors in humoral immunity. In individuals with CRC, the B cell populations found in peripheral blood, mesenteric LN, and at the site of the primary tumor differ markedly from those in healthy subjects, suggesting an activation of B cells within the TME [42]. Notably, in cases of metastatic CRC, there is a substantial decrease in B cell counts [43]. B cells are diverse, with subsets such as IgA + IGLC2+ plasma cells being correlated with an unfavorable outcome in CRC patients. On the other hand, the presence of vigorously dividing IGLC2 plasma cells and circulating B cells is indicative of a more positive prognosis [44].

## 3. CRC and TLS

### 3.1. Formation of TLSs

TLS are immune cell conglomerates that form in tissues typically not involved in lymphatic function, often in the context of ongoing inflammation. This can be triggered by a range of conditions such as persistent viral infections, autoimmune reactions, following tissue grafts, or in the presence of cancer [4,45,46]. TLS exhibit architectural and operational parallels with SLOs, exemplified by the presence of a T cell region populated by mature DCs, germinal centers (GCs) teeming with FDCs, along with active B cell proliferation and the presence of HEVs [3]. As TLS develop, both the B and T cell regions undergo enhancement with an increase in FDCs [47]. Unlike SLOs, NK are not present in TLS [48]. TLS is present in various types of solid tumors and is generally associated with improved prognosis [8–10]. TLS has been found in the mucosa of CRC patients, and pathological studies have shown that TLS in the mucosa of CRC patients exhibits a highly organized structure with clear T cell and B cell zones, indicating that it may maintain an effective immune response [49]. In colon inflammation-related cancer models, the expansion of lymphoid tissue during the process of inflammation-driven polyp formation suggests that it may maintain inflammation or help suppress anti-tumor immune responses [49].

**Figure 1.**
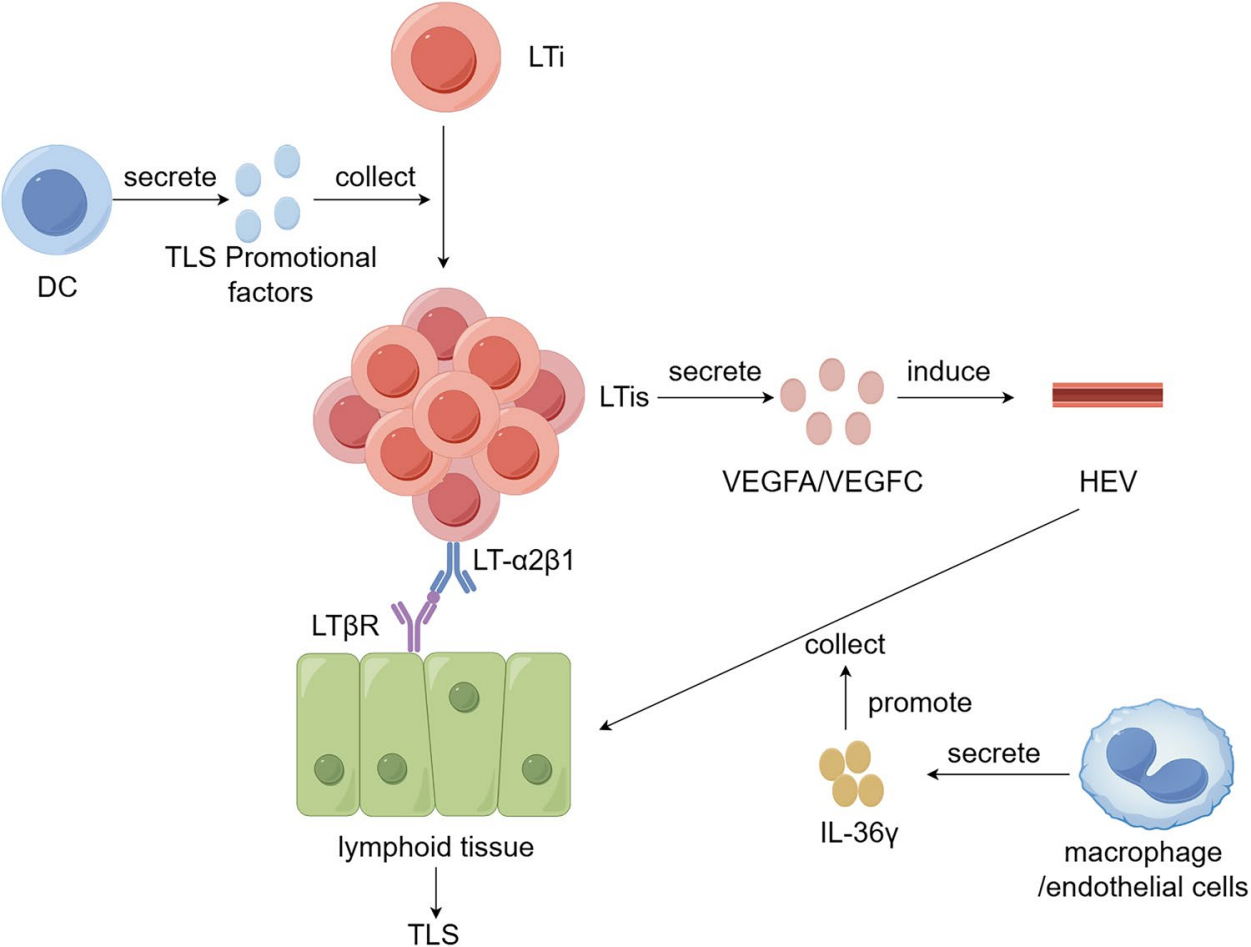
Formation of TLSs. DCs release promotional factors that collect LTi cells to the site of injury, where LTi cells express LT-α2β1 and interact with LTβR on lymphoid tissue. Upon interaction, LTi cells secrete VEGFA/VEGFC and induce HEV formation. Furthermore, the cytokine IL-36γ secreted by macrophages and endothelial cells enhances HEV-mediated lymphocyte recruitment, leading to the development of a tertiary lymphoid structure. Draw by Figdraw.

SLOs, which include the spleen, LN, tonsils, Peyer’s patches, and mucosa-associated lymphoid tissues, develop through a complex interplay between lymphoid tissue- inducer (LTi) cells and stromal elements. Akin to SLOs, the emergence of TLS is orchestrated by a network of chemokines, cytokines, and molecules that facilitate cell adhesion. The initial phase of TLS development is significantly influenced by the aberrant expression of dendritic cell-secreted factors [50]. These factors that encourage TLS formation are instrumental in drawing LTi cells to the affected area [51]. LTi cells express the lymphotoxin α1β2 ligand (LTα1β2) on their surface, which interacts with the lymphotoxin β receptor (LTβR) present on lymphoid tissues and stromal cells. Upon this interaction with LTi cells, the secretion of vascular endothelial growth factors, particularly vascular endothelial growth factor A (VEGFA) and vascular endothelial growth factor C (VEGFC), is triggered, leading to the genesis of high endothelial venules (HEVs) [52]. Other vascular adhesion molecules, including vascular cell adhesion molecule 1 (VCAM1) and mucosal vascular addressin cell adhesion molecule 1 (MADCAM1), play a role in attracting immune cells to TLS and in their subsequent retention [52]. Furthermore, the secretion of the cytokine IL-36γ by macrophages and endothelial cells enhances the expression of VCAM1 and intercellular adhesion molecule 1 (ICAM-1) on stromal and vascular endothelial cells. This, in conjunction with chemokines such as chemokine (C-C motif) ligand 2 (CCL2) and chemokine (C-C motif) ligand 20 (CCL20), augments the capacity of HEV to recruit lymphocytes, thus fostering the development and maturation of TLS within the context of human CRC [53,54] (Figure 1).

### 3.2. Features and functions of TLS

The T cell zone of TLS is mainly composed of T cells, including CD8+ cytotoxic T cells, CD4+ Th1 cells, and regulatory T cells [26]. The density of T cells is considered a prognostic marker for CRC patients [55]. Previous studies have found that the number of T cells in TLS significantly increases during inflammation-driven colon cancer, indicating that the occurrence of TLS is related to T cell infiltration and has potential prognostic value [10]. One study found that the density of Th cells (especially the Th2 effector subgroup) and macrophages in TLS of recurrent patients was significantly higher than that of non-recurrent patients, and a higher proportion of Th cells may be an independent risk factor for recurrence [56]. Another study found that plasmacytoid DCs were present in TLS associated with colon cancer and were preferentially located near CD4 T cells in the T cell zone [57]. The presence of a large number of activated phenotypes of plasmacytoid DCs suggests that CD4 effector T cells may promote anti-tumor immunity by enhancing the ability of DCs to induce CD8 T cell responses and directly promoting the expansion of CD8 T cells [57,58]. Helicobacter hepaticus (Hhep) is an adherent bacterium primarily found in the cecum and colon. It is known for inducing local immune responses, which vary depending on the immune status of the host. A recent study found that Hhep colonization can induce the generation of Hhep-specific Tfh and support the maturation of tertiary lymphoid structures adjacent to Hhep + tumors [55]. This result suggests that Hhep-specific Tfh cells have the potential to promote TLS and anti-tumor immunity, providing a new approach for future anti-tumor immune therapy for CRC.

Historically, B lymphocytes have been acknowledged for their central role in humoral immunity and their contribution to combating tumors. Recent research has shed light on the regulatory function of B cells within the immune system’s reaction to cancerous growths and lymphoid cancers [59]. The prevailing view is that B cells are pivotal in the production of antibodies instrumental in the destruction of malignant cells through mechanisms such as antibody-dependent cellular cytotoxicity and phagocytic processes [60]. Emerging findings on tumor-associated TLS indicate that B cells within these structures correlate with improved patient outcomes and a positive response to immunotherapeutic interventions. Within TLS, B cells undergo differentiation and maturation, serving as antigen-presenting entities that engage CD8+ T cells, thereby amplifying an immune reaction [61]. CD20+ B cells, predominant in the B cell zones of TLS, are noted for their substantial presence in CRC and are linked to enhanced prognoses [62]. An elevated IgG ratio has been correlated with heightened clonality and extensive mutation within tumor tissues [63]. Research has pinpointed IgG plasma cells predominantly in areas external to TLS and tumor stroma in the CRC TME, suggesting that CD20+ B cells may evolve into IgG plasma cells, relocating to the tumor stroma to generate antibodies that bolster anti-tumor immunity [25]. CD86+ B cells have been shown to increase in the TME of CRC [64]. B cells with strong antigen-presenting ability are enriched in the CD21- subset [65]. A recent study found a physiologically relevant B cell subset BAPC with immunostimulatory properties, which has strong antigen-presenting ability, characterized by upregulation of CD86 and downregulation of CD21[66]. A study found that BAPCs located in lymphoid follicles in TLS co-localize with T cells in TLS, and the density of BAPCs increases in high TLS samples [67]. Further *in vitro* experiments have shown that BAPCs induce tumor antigen-specific responses in autologous T cells of CRC patients, demonstrating the antigen-presenting ability of BAPCs in cancer patients and the potential to induce anti-tumor T cell responses [67]. CD68 macrophages in TLS are called tingible body macrophages and act as scavengers of apoptotic lymphocytes [68]. A study found that the density of CD68 macrophages in recurrent patients significantly increased, indicating that a higher tingible body macrophages density may be an independent risk factor for recurrence [56].

HEV, integral to the composition of tertiary lymphoid structures, play a pivotal role in the process of immune cell extravasation from the bloodstream, as well as in the release of various chemotactic cytokines, including CCL19, CCL21, CXCL10, CXCL12, and CXCL13. These venules have the capacity to present peripheral lymph node addressin, which is the binding partner for the L-selectin/CD62L complex on leukocytes, facilitating the infiltration of immune cells and promoting anti-tumor activities [69,70]. A wealth of research has demonstrated a positive correlation between the presence of HEV and favorable clinical prognoses across a spectrum of cancer types, such as in cases of breast, melanoma, pancreatic, brain, and oral squamous cell carcinomas [71–75]. In contrast to their absence in healthy colonic tissue, HEVs have been identified at the periphery of tumor infiltrates in CRC, where their presence is indicative of an adverse prognosis [76,77]. Although they are distributed differently, HEV in the tumor and peritumor areas have no morphological differences [78]. HEV is also a prognostic biomarker in CRC. It depends on the presence of tumor-infiltrating lymphocytes [10]. HEV is a vessel used for lymphocyte transport, and MSI CRC has significantly higher HEV density than MSS CRC [78,79]. Recent studies have shown that elevated HEV/TLS can reduce TNM stage, prolong OS and DFS, and recruit more immune cells, such as CD3+ T cells, CD8+ T cells and M1 macrophages, thereby improving the tumor immune microenvironment [80]. IL-36γ is an IL-1 family cytokine that participates in the polarization of type 1 immune responses [81]. Previous studies have found that high levels of IL-36γ production are correlated with low survival rates in CRC patients [82]. In a mouse model of colon cancer, Weinstein et al. supported this conclusion and found that the main cells expressing IL-36γ in TLS were HEV-related VECs and observed that HEV controlled the formation of TLS by producing IL-36γ [54].

NKp44 + ILC3 is the most common ILC subset in the human intestine [83]. Atsuyo et al. found that NKp44 + ILC3 exists in the tumor tissue of CRC and decreases with tumor progression [84]. Further studies have shown that the decrease in the number of NKp44 + ILC3 is accompanied by a decrease in TLS density and the expression of genes related to lymphoid structure formation in these cells [84]. This finding reveals the role of ILC in the tumor immune microenvironment of CRC, and further research is needed to investigate the significance of ILC-induced TLS in anti-tumor therapy for CRC. Chemokines are a large family of cytokines that can induce immune cells in the TME to regulate the host’s immune response to cancer [85]. The effect of 12-chemokine features (CCL-2, −3, −4, −5, −8, −18, −19, −21, CXCL-9, −10, −11, −13) as a predictor of TLS expression has been validated in CRC [86]. A study conducted an examination of a database comprising 975 cases of resected CRC from three distinct cohorts in France, Japan, and the United States. The findings indicated that an elevated status of a 12-chemokine signature was linked to a reduced rate of recurrence, an increased expression of local TLS, and several key characteristics of CRC, including tumors located on the right side, BRAF gene mutations, high CpG island methylator phenotype status, and high microsatellite instability status. These results imply that the 12-chemokine signature status might be indicative of the host’s capacity to oversee the immune response to CRC, and it appears to be separate from the progression state of the tumor [87].

### 3.3. Patient prognosis

The concept of Crohn’s-like lymphoid reaction (CLR) is characterized by dense lymphoid aggregates at the invasive margin of tumors, which typically lack the classic germinal centers and are considered unorganized lymphoid infiltrates. The assessment of CLR in H&E (hematoxylin and eosin)-stained sections has been proven to be a valuable method for evaluating the immune response in CRC [88]. The presence of CLR can serve as an indirect indicator, suggesting immune activity within the tumor microenvironment and potentially indicating the presence of TLS. Therefore, TLS can be assessed using H&E-stained sections, providing an accessible routine method for histopathological evaluation. However, as noted in a study, qualitative assessments of TLSs may suffer from observer variability, whereas quantitative methods, which focus on the density and distribution of TLS, have shown stronger correlations with patient outcomes, providing greater prognostic value [89].

Compared to H&E staining, immunohistochemistry offers the ability to finely differentiate between various cell types within TLSs, allowing for a more detailed understanding of the specific roles different immune cell types play in the tumor immune response. Based on this approach, to better study the association between the structure of TLS and CRC prognosis, some scholars have proposed different classifications. Yamaguchi et al. analyzed 67 TLS found in 353 CRC patients and divided TLS in CRC tissue into five types based on the density of cell components: GC-rich TLS, T-helper (Th)-cell-rich TLS, B-cell-rich TLS, FDC-rich TLS, and CTL/B/Th-TLS. They found that the density of Th-rich type was significantly associated with disease recurrence, while the density of immune cells composed of GC B cells and Tfh cells was not related to prognosis [56]. Posch et al. reviewed 109 non-metastatic stage II and III CRC patients and divided TLS into three mature subtypes based on FDC and GC: early TLS (E-TLS, undifferentiated FDC), primary follicular-like TLS (PFL-TLS, FDC present but no GC), and secondary follicular-like TLS (SFL-TLS, with GC). They subsequently confirmed that low SFL-TLS was associated with an increased risk of recurrence, indicating that the degree of TLS maturity has significant prognostic and potential predictive value [90]. The proportion of tumor stroma (TSP) is closely related to the prognosis of certain cancer patients, with a high TSP (greater than 50%) linked to advanced T stage, lymph node metastasis, vascular invasion, a higher rate of recurrence, and a shorter lifespan [91,92]. Wang and colleagues integrated TSP with the classification system suggested by Posch and his team, positing that a high density of P-TLS and a low TSP (L-TSP) are independent and advantageous prognostic indicators for patients with non-metastatic CRC [93].

Salama and colleagues examined the prevalence of FoxP3+ cells within TLS in stage II colon cancer, identifying a high concentration of these cells in TLS as an unfavorable prognostic indicator for overall survival [94]. Thomas et al. analyzed the presence and composition of TLS in CRC lung metastasis and demonstrated that high levels of CD8+ cells and high CD8/FoxP3 ratio in TLS were positively correlated with OS of patients after CRC lung metastasis resection. The balance between CD8+ and FoxP3+ cells in TLS appears to be a key factor for the survival of CRC patients [95]. Zhang and colleagues conducted an analysis of the occurrence and characteristics of TLS in liver metastases of CRC, discovering a significant correlation between the spread and quantity of TLS and the relapse-free survival and overall survival of patients with CRC liver metastasis. Utilizing the distribution and prevalence of TLS, they developed an immune scoring system that integrates the T score and P score to forecast the prognosis of CRC liver metastasis patients [96].

## 4. Potential clinical applications

The formation of TLS is associated with good patient prognosis and therefore has potential applications in anti-tumor therapy. In CRC, T-bet expressing DCs have been used to induce T lymphocyte infiltration and TLS formation [97]. This anti-tumor effect is dependent on IL-36γ and can be inhibited by IL-36 receptor antagonists or by a deficiency of the IL-36 receptor.

ICIs are able to induce the formation of TLS in the TME, which have an anti-tumor effect [98]. According to the latest research results released at the annual meeting of the Cancer Immunotherapy Society, PD-L1 targeted drugs combined with multi-kinase inhibitors with anti-angiogenic activity have produced clinical responses in TLS-positive tumor patients, even in cancer types that are usually considered resistant to checkpoint inhibitors [99]. This result confirms the potential of tertiary lymphoid structures as a predictive biomarker for drug response based on immune checkpoint inhibitors. A study found that in patients with renal cell carcinoma receiving immune checkpoint inhibitor therapy, treatment response and progression-free survival were associated with IgG staining of tumor cells, indicating that intratumorally TLS is associated with immune therapy response [100]. For CRC, a retrospective analysis found that high expression of programmed cell death receptor 1 at the invasive margin was significantly associated with the presence of TLS [101]. Therefore, immune checkpoint inhibitors related to TLS may be an effective treatment for CRC, and it is foreseeable that this will be a hot direction for CRC immunotherapy.

There is growing evidence that metformin can reduce the incidence and mortality from CRC. Saito found that metformin treatment significantly increased the density of TLS in the tumor stroma of CRC patients, indicating that metformin may induce TLS to increase the limited lymphatic spread of tumor cells with anti-tumor properties of TIL [102]. This result reveals a new application of metformin in CRC. However, this conclusion needs further research to confirm.

## 5. Summary and discussion

In summary, CRC-related TLS occurs in localized areas of the tumor and is an important component of anti-tumor immunity in the TME. Current research suggests that the related components of TLSs, such as HEV, B-cell zone, T-cell zone, and related factors, are associated with CRC prognosis, but their contributions and related mechanisms have only recently been preliminarily understood and require further in-depth research. For instance, although most studies present IL-36γ as a positive prognostic factor in CRC, conflicting reports suggest it may correlate with lower survival rates in certain contexts. This discrepancy highlights the complexity of IL-36γ's role, which may vary depending on specific TME, necessitating further studies to clarify its prognostic significance.

The presence of TLSs generally correlates with a favorable prognosis in CRC, and improved classification systems could enhance predictions and mitigate risks related to metastasis and recurrence. Moreover, recent clinical trial outcomes have underscored the potential of TLSs as predictive biomarkers for drug response, particularly in the context of immune checkpoint inhibitors. However, these findings still require validation through further clinical trials.

Currently, the detection of TLSs primarily relies on multiplex immunohistochemistry or immunofluorescence to stain immune cell lineage markers within tissue samples. Recent studies have also utilized machine learning to quantify CLR density in whole-slide images of HE-stained sections, demonstrating that fully automated CLR density quantification serves as an independent prognostic factor (discovery cohort HR: 0.58, validation cohort HR: 0.45). High CLR density has been associated with improved overall survival [103]. Additionally, new machine learning models capable of automatically detecting, counting, and classifying TLS in HE-stained sections have been developed. Importantly, the TLS score derived from these models functions independently of traditional tumor prognostic factors such as staging and grading, suggesting that integrating this score into current evaluation processes could refine staging systems and improve risk stratification [104].

Despite these advancements, IHC remains invaluable in TLS research. The specific identification of cell types *via* IHC is crucial for a deeper understanding of TLS function within immune responses. Moreover, assessing the functional status of cells through IHC is essential for evaluating the activity of tumor immune evasion mechanisms. IHC also facilitates quantitative analysis and pathway studies, which are key to understanding TLS roles across different tumor environments. Furthermore, the high-resolution spatial information provided by IHC is indispensable for studying TLS within the context of spatial omics.

In conclusion, while the integration of machine learning with traditional staining methods has expanded the toolkit for TLS analysis, the irreplaceable contributions of IHC in cell type specificity, functional analysis, and spatial resolution ensure its continued relevance in both current and future TLS research.

## Data Availability

Anonymized data are available upon reasonable request.

## References

[1] Boehm T, Bleul CC. The evolutionary history of lymphoid organs. Nat Immunol. 2007;8(2):131–135. doi:10.1038/ni1435. 17242686

[2] Boehm T, Hess I, Swann JB. Evolution of lymphoid tissues. Trends Immunol. 2012;33(6):315–321. doi:10.1016/j.it.2012.02.005. 22483556

[3] Rodriguez AB, Engelhard VH. Insights into tumor-associated tertiary lymphoid structures: novel targets for antitumor immunity and cancer immunotherapy. Cancer Immunol Res. 2020;8(11):1338–1345. doi:10.1158/2326-6066.CIR-20-0432. 33139300 PMC7643396

[4] Sautès-Fridman C, Petitprez F, Calderaro J, et al. Tertiary lymphoid structures in the era of cancer immunotherapy. Nat Rev Cancer. 2019;19(6):307–325. doi:10.1038/s41568-019-0144-6. 31092904

[5] Sautès-Fridman C, Cherfils-Vicini J, Damotte D, et al. Tumor microenvironment is multifaceted. Cancer Metastasis Rev. 2011;30(1):13–25. doi:10.1007/s10555-011-9279-y. 21271351

[6] Quail DF, Joyce JA. Microenvironmental regulation of tumor progression and metastasis. Nat Med. 2013;19(11):1423–1437. doi:10.1038/nm.3394. 24202395 PMC3954707

[7] Paijens ST, Vledder A, de Bruyn M, et al. Tumor-infiltrating lymphocytes in the immunotherapy era. Cell Mol Immunol. 2021;18(4):842–859. doi:10.1038/s41423-020-00565-9. 33139907 PMC8115290

[8] Calderaro J, Petitprez F, Becht E, et al. Intra-tumoral tertiary lymphoid structures are associated with a low risk of early recurrence of hepatocellular carcinoma. J Hepatol. 2019;70(1):58–65. doi:10.1016/j.jhep.2018.09.003. 30213589

[9] Dieu-Nosjean MC, Antoine M, Danel C, et al. Long-term survival for patients with non-small-cell lung cancer with intratumoral lymphoid structures. J Clin Oncol. 2008;26(27):4410–4417. doi:10.1200/JCO.2007.15.0284. 18802153

[10] Di Caro G, Bergomas F, Grizzi F, et al. Occurrence of tertiary lymphoid tissue is associated with T-cell infiltration and predicts better prognosis in early-stage colorectal cancers. Clin Cancer Res. 2014;20(8):2147–2158. doi:10.1158/1078-0432.CCR-13-2590. 24523438

[11] Gu-Trantien C, Loi S, Garaud S, et al. CD4^+^ follicular helper T cell infiltration predicts breast cancer survival. J Clin Invest. 2013;123(7):2873–2892. doi:10.1172/JCI67428. 23778140 PMC3696556

[12] Sung H, Ferlay J, Siegel RL, et al. Global Cancer Statistics 2020: GLOBOCAN estimates of incidence and mortality worldwide for 36 cancers in 185 countries. CA Cancer J Clin. 2021;71(3):209–249. doi:10.3322/caac.21660. 33538338

[13] Schmitt M, Greten FR. The inflammatory pathogenesis of colorectal cancer. Nat Rev Immunol. 2021;21(10):653–667. doi:10.1038/s41577-021-00534-x. 33911231

[14] Catalano V, Turdo A, Di Franco S, et al. Tumor and its microenvironment: a synergistic interplay. Semin Cancer Biol. 2013;23(6 Pt B):522–532. doi:10.1016/j.semcancer.2013.08.007. 24012661

[15] Tabuso M, Homer-Vanniasinkam S, Adya R, et al. Role of tissue microenvironment resident adipocytes in colon cancer. World J Gastroenterol. 2017;23(32):5829–5835. doi:10.3748/wjg.v23.i32.5829 28932075 PMC5583568

[16] Roma-Rodrigues C, Mendes R, Baptista PV, et al. Targeting tumor microenvironment for cancer therapy. Int J Mol Sci. 2019;20(4):840. doi:10.3390/ijms20040840. 30781344 PMC6413095

[17] Mizuno R, Kawada K, Itatani Y, et al. The role of tumor-associated neutrophils in colorectal cancer. Int J Mol Sci. 2019;20(3):529. doi:10.3390/ijms20030529. 30691207 PMC6386937

[18] Mizuno R, Kawada K, Sakai Y. Prostaglandin E2/EP signaling in the tumor microenvironment of colorectal cancer. Int J Mol Sci. 2019;20(24):6254. doi:10.3390/ijms20246254. 31835815 PMC6940958

[19] Chiba F, Soda K, Yamada S, et al. The importance of tissue environment surrounding the tumor on the development of cancer cachexia. Int J Oncol. 2014;44(1):177–186. doi:10.3892/ijo.2013.2180. 24247481

[20] Grivennikov SI, Greten FR, Karin M. Immunity, inflammation, and cancer. Cell. 2010;140(6):883–899. doi:10.1016/j.cell.2010.01.025. 20303878 PMC2866629

[21] Hiratsuka S, Nakamura K, Iwai S, et al. MMP9 induction by vascular endothelial growth factor receptor-1 is involved in lung-specific metastasis. Cancer Cell. 2002;2(4):289–300. doi:10.1016/s1535-6108(02)00153-8. 12398893

[22] Lin EY, Nguyen AV, Russell RG, et al. Colony-stimulating factor 1 promotes progression of mammary tumors to malignancy. J Exp Med. 2001;193(6):727–740. doi:10.1084/jem.193.6.727. 11257139 PMC2193412

[23] Zhou F, Feng B, Yu H, et al. Tumor microenvironment-activatable prodrug vesicles for nanoenabled cancer chemoimmunotherapy combining immunogenic cell death induction and CD47 blockade. Adv Mater. 2019;31(14):e1805888. doi:10.1002/adma.201805888. 30762908

[24] Schumacher TN, Thommen DS. Tertiary lymphoid structures in cancer. Science. 2022;375(6576):eabf9419. doi:10.1126/science.abf9419. 34990248

[25] Senovilla L, Vacchelli E, Galon J, et al. Trial watch: prognostic and predictive value of the immune infiltrate in cancer. Oncoimmunology. 2012;1(8):1323–1343. doi:10.4161/onci.22009. 23243596 PMC3518505

[26] Lee N, Zakka LR, Mihm MC, Jr, et al. Tumour-infiltrating lymphocytes in melanoma prognosis and cancer immunotherapy. Pathology. 2016;48(2):177–187. doi:10.1016/j.pathol.2015.12.006. 27020390

[27] Oliveira G, Wu CJ. Dynamics and specificities of T cells in cancer immunotherapy. Nat Rev Cancer. 2023;23(5):295–316. doi:10.1038/s41568-023-00560-y. 37046001 PMC10773171

[28] Aristin Revilla S, Kranenburg O, Coffer PJ. Colorectal cancer-infiltrating regulatory T cells: functional heterogeneity, metabolic adaptation, and therapeutic targeting. Front Immunol. 2022;13:903564. doi:10.3389/fimmu.2022.903564. 35874729 PMC9304750

[29] Hiam-Galvez KJ, Allen BM, Spitzer MH. Systemic immunity in cancer. Nat Rev Cancer. 2021;21(6):345–359. doi:10.1038/s41568-021-00347-z. 33837297 PMC8034277

[30] Bruni D, Angell HK, Galon J. The immune contexture and Immunoscore in cancer prognosis and therapeutic efficacy. Nat Rev Cancer. 2020;20(11):662–680. doi:10.1038/s41568-020-0285-7. 32753728

[31] Camus M, Tosolini M, Mlecnik B, et al. Coordination of intratumoral immune reaction and human colorectal cancer recurrence. Cancer Res. 2009;69(6):2685–2693. doi:10.1158/0008-5472.CAN-08-2654. 19258510

[32] Speiser DE, Chijioke O, Schaeuble K, et al. CD4 T cells in cancer. Nat Cancer. 2023;4(3):317–329. doi:10.1038/s43018-023-00521-2+. 36894637

[33] Schmitt N, Ueno H. Regulation of human helper T cell subset differentiation by cytokines. Curr Opin Immunol. 2015;34:130–136. doi:10.1016/j.coi.2015.03.007. 25879814 PMC4465198

[34] Müller-Hermelink N, Braumüller H, Pichler B, et al. TNFR1 signaling and IFN-gamma signaling determine whether T cells induce tumor dormancy or promote multistage carcinogenesis. Cancer Cell. 2008;13(6):507–518. doi:10.1016/j.ccr.2008.04.001. 18538734

[35] Braumüller H, Mauerer B, Andris J, et al. The cytokine network in colorectal cancer: implications for new treatment strategies. Cells. 2022;12(1):138. doi:10.3390/cells12010138. 36611932 PMC9818504

[36] Robert C, Thomas L, Bondarenko I, et al. Ipilimumab plus Dacarbazine for previously untreated metastatic Melanoma. N Engl J Med. 2011;364(26):2517–2526. doi:10.1056/NEJMoa1104621. 21639810

[37] Amicarella F, Muraro MG, Hirt C, et al. Dual role of tumour-infiltrating T helper 17 cells in human colorectal cancer. Gut. 2017;66(4):692–704. doi:10.1136/gutjnl-2015-310016. 26719303 PMC5529969

[38] Galon J, Costes A, Sanchez-Cabo F, et al. Type, density, and location of immune cells within human colorectal tumors predict clinical outcome. Science. 2006;313(5795):1960–1964. doi:10.1126/science.1129139. 17008531

[39] Saito T, Nishikawa H, Wada H, et al. Two FOXP3 + CD4 + T cell subpopulations distinctly control the prognosis of colorectal cancers. Nat Med. 2016;22(6):679–684. doi:10.1038/nm.4086. 27111280

[40] Schürch CM, Bhate SS, Barlow GL, et al. Coordinated cellular neighborhoods orchestrate antitumoral immunity at the colorectal cancer invasive front. Cell. 2020;182(5):1341–1359.e19. doi:10.1016/j.cell.2020.07.005. 32763154 PMC7479520

[41] Niogret J, Berger H, Rebe C, et al. Follicular helper-T cells restore CD8+-dependent antitumor immunity and anti-PD-L1/PD-1 efficacy. J Immunother Cancer. 2021;9(6):e002157. doi:10.1136/jitc-2020-002157. 34103351 PMC8190041

[42] Zirakzadeh AA, Marits P, Sherif A, et al. Multiplex B cell characterization in blood, lymph nodes, and tumors from patients with malignancies. J Immunol. 2013;190(11):5847–5855. doi:10.4049/jimmunol.1203279. 23630345

[43] Shimabukuro-Vornhagen A, Schlößer HA, Gryschok L, et al. Characterization of tumor-associated B-cell subsets in patients with colorectal cancer. Oncotarget. 2014;5(13):4651–4664. doi:10.18632/oncotarget.1701. 25026291 PMC4148088

[44] Wang W, Zhong Y, Zhuang Z, et al. Multiregion single-cell sequencing reveals the transcriptional landscape of the immune microenvironment of colorectal cancer. Clin Transl Med. 2021;11(1):e253. doi:10.1002/ctm2.253. 33463049 PMC7775989

[45] Neyt K, Perros F, GeurtsvanKessel CH, et al. Tertiary lymphoid organs in infection and autoimmunity. Trends Immunol. 2012;33(6):297–305. doi:10.1016/j.it.2012.04.006. 22622061 PMC7106385

[46] Pipi E, Nayar S, Gardner DH, et al. Tertiary lymphoid structures: autoimmunity goes local. Front Immunol. 2018;9:1952. doi:10.3389/fimmu.2018.01952. 30258435 PMC6143705

[47] Siliņa K, Soltermann A, Attar FM, et al. Germinal centers determine the prognostic relevance of tertiary lymphoid structures and are impaired by corticosteroids in lung squamous cell carcinoma. Cancer Res. 2018;78(5):1308–1320. doi:10.1158/0008-5472.CAN-17-1987. 29279354

[48] Platonova S, Cherfils-Vicini J, Damotte D, et al. Profound coordinated alterations of intratumoral NK cell phenotype and function in lung carcinoma. Cancer Res. 2011;71(16):5412–5422. doi:10.1158/0008-5472.CAN-10-4179. 21708957

[49] Bergomas F, Grizzi F, Doni A, et al. Tertiary intratumor lymphoid tissue in colo-rectal cancer. Cancers (Basel). 2011;4(1):1–10. doi:10.3390/cancers4010001. 24213222 PMC3712686

[50] Luo S, Zhu R, Yu T, et al. Chronic inflammation: a common promoter in tertiary lymphoid organ neogenesis. Front Immunol. 2019;10:2938. doi:10.3389/fimmu.2019.02938. 31921189 PMC6930186

[51] Denton AE, Innocentin S, Carr EJ, et al. Type I interferon induces CXCL13 to support ectopic germinal center formation. J Exp Med. 2019;216(3):621–637. doi:10.1084/jem.20181216. 30723095 PMC6400543

[52] N J, J T, Sl N, et al. Tertiary lymphoid structures and B lymphocytes in cancer prognosis and response to immunotherapies. Oncoimmunology. 2021;10(1):1900508. doi:10.1080/2162402X.2021.1900508. 33854820 PMC8018489

[53] Bridgewood C, Stacey M, Alase A, et al. IL-36gamma has proinflammatory effects on human endothelial cells. Exp Dermatol. 2017;26(5):402–408. doi:10.1111/exd.13228. 27673278

[54] Weinstein AM, Giraldo NA, Petitprez F, et al. Association of IL-36gamma with tertiary lymphoid structures and inflammatory immune infiltrates in human colorectal cancer. Cancer Immunol Immunother. 2019;68(1):109–120. doi:10.1007/s00262-018-2259-0. 30315348 PMC7185158

[55] Overacre-Delgoffe AE, Bumgarner HJ, Cillo AR, et al. Microbiota-specific T follicular helper cells drive tertiary lymphoid structures and anti-tumor immunity against colorectal cancer. Immunity. 2021;54(12):2812–2824.e4. doi:10.1016/j.immuni.2021.11.003. 34861182 PMC8865366

[56] Yamaguchi K, Ito M, Ohmura H, et al. Helper T cell-dominant tertiary lymphoid structures are associated with disease relapse of advanced colorectal cancer. Oncoimmunology. 2020;9(1):1724763. doi:10.1080/2162402X.2020.1724763. 32117589 PMC7028340

[57] Kießler M, Plesca I, Sommer U, et al. Tumor-infiltrating plasmacytoid dendritic cells are associated with survival in human colon cancer. J Immunother Cancer. 2021;9(3):e001813. doi:10.1136/jitc-2020-001813. 33762320 PMC7993360

[58] Borst J, Ahrends T, Bąbała N, et al. CD4 T cell help in cancer immunology and immunotherapy. Nat Rev Immunol. 2018;18(10):635–647. doi:10.1038/s41577-018-0044-0. 30057419

[59] Sarvaria A, Madrigal JA, Saudemont A. B cell regulation in cancer and anti-tumor immunity. Cell Mol Immunol. 2017;14(8):662–674. doi:10.1038/cmi.2017.35. 28626234 PMC5549607

[60] Franchina DG, Grusdat M, Brenner D. B-cell metabolic remodeling and cancer. Trends Cancer. 2018;4(2):138–150. doi:10.1016/j.trecan.2017.12.006. 29458963

[61] Yamakoshi Y, Tanaka H, Sakimura C, et al. Immunological potential of tertiary lymphoid structures surrounding the primary tumor in gastric cancer. Int J Oncol. 2020;57(1):171–182. doi:10.3892/ijo.2020.5042. 32319601 PMC7252463

[62] Edin S, Kaprio T, Hagström J, et al. The prognostic importance of CD20+ B lymphocytes in colorectal cancer and the relation to other immune cell subsets. Sci Rep. 2019;9(1):19997. Published 2019 Dec 27. doi:10.1038/s41598-019-56441-8. 31882709 PMC6934737

[63] Breedveld A, van Egmond M. IgA and FcαRI: pathological roles and therapeutic opportunities. Front Immunol. 2019;10:553. doi:10.3389/fimmu.2019.00553. 30984170 PMC6448004

[64] Lu C, Schardey J, Wirth U, et al. Analysis of circulating immune subsets in primary colorectal cancer. Cancers. 2022;14(24):6105. doi:10.3390/cancers14246105. 36551592 PMC9776578

[65] Bruno TC, Ebner PJ, Moore BL, et al. Antigen-presenting intratumoral B cells affect CD4 TIL phenotypes in non-small cell lung cancer patients. Cancer Immunol Res. 2017;5(10):898–907. doi:10.1158/2326-6066.CIR-17-0075+. 28848053 PMC5788174

[66] Shimabukuro-Vornhagen A, García-Márquez M, Fischer RN, et al. Antigen-presenting human B cells are expanded in inflammatory conditions. J Leukoc Biol. 2017;101(2):577–587. doi:10.1189/jlb.5A0416-182R. 27534894

[67] Wennhold K, Thelen M, Lehmann J, et al. CD86+ antigen-presenting B cells are increased in cancer, localize in tertiary lymphoid structures, and induce specific T-cell responses. Cancer Immunol Res. 2021;9(9):1098–1108. doi:10.1158/2326-6066.CIR-20-0949. 34155067

[68] Teillaud JL, Dieu-Nosjean MC. Tertiary lymphoid structures: an anti-tumor school for adaptive immune cells and an antibody factory to fight cancer? Front Immunol. 2017;8:830. doi:10.3389/fimmu.2017.00830. 28785261 PMC5519532

[69] Ivetic A, Hoskins Green HL, Hart SJ. L-selectin: a major regulator of leukocyte adhesion, migration and signaling. Front Immunol. 2019;10:1068. doi:10.3389/fimmu.2019.01068. 31139190 PMC6527602

[70] Girard JP, Moussion C, Förster R. HEVs, lymphatics and homeostatic immune cell trafficking in lymph nodes. Nat Rev Immunol. 2012;12(11):762–773. doi:10.1038/nri3298. 23018291

[71] Martinet L, Garrido I, Filleron T, et al. Human solid tumors contain high endothelial venules: association with T- and B-lymphocyte infiltration and favorable prognosis in breast cancer. Cancer Res. 2011;71(17):5678–5687. doi:10.1158/0008-5472.CAN-11-0431. 21846823

[72] Martinet L, Le Guellec S, Filleron T, et al. High endothelial venules (HEVs) in human melanoma lesions: major gateways for tumor-infiltrating lymphocytes. Oncoimmunology. 2012;1(6):829–839. doi:10.4161/onci.20492. 23162750 PMC3489738

[73] Bahmani B, Uehara M, Ordikhani F, et al. Ectopic high endothelial venules in pancreatic ductal adenocarcinoma: a unique site for targeted delivery. EBioMedicine. 2018;38:79–88. doi:10.1016/j.ebiom.2018.11.030. 30497977 PMC6306381

[74] He B, Jabouille A, Steri V, et al. Vascular targeting of LIGHT normalizes blood vessels in primary brain cancer and induces intratumoural high endothelial venules. J Pathol. 2018;245(2):209–221. doi:10.1002/path.5080. 29603739 PMC6737176

[75] Wirsing AM, Ervik IK, Seppola M, et al. Presence of high-endothelial venules correlates with a favorable immune microenvironment in oral squamous cell carcinoma. Mod Pathol. 2018;31(6):910–922. doi:10.1038/s41379-018-0019-5. 29416107

[76] Dieu-Nosjean MC, Goc J, Giraldo NA, et al. Tertiary lymphoid structures in cancer and beyond. Trends Immunol. 2014;35(11):571–580. doi:10.1016/j.it.2014.09.006. 25443495

[77] Bento DC, Jones E, Junaid S, et al. High endothelial venules are rare in colorectal cancers but accumulate in extra-tumoral areas with disease progression. Oncoimmunology. 2015;4(3):e974374. Published 2015 Apr 2. doi:10.4161/2162402X.2014.974374. 25949892 PMC4404788

[78] Pfuderer PL, Ballhausen A, Seidler F, et al. High endothelial venules are associated with microsatellite instability, hereditary background, and immune evasion in colorectal cancer. Br J Cancer. 2019;121(5):395–404. doi:10.1038/s41416-019-0514-6. 31358939 PMC6738093

[79] Ager A, May MJ. Understanding high endothelial venules: lessons for cancer immunology. Oncoimmunology. 2015;4(6):e1008791. doi:10.1080/2162402X.2015.1008791. 26155419 PMC4485764

[80] Zhan Z, Shi-Jin L, Yi-Ran Z, et al. High endothelial venules proportion in tertiary lymphoid structure is a prognostic marker and correlated with anti-tumor immune microenvironment in colorectal cancer. Ann Med. 2023;55(1):114–126. doi:10.1080/07853890.2022.2153911. 36503344 PMC9754014

[81] Vigne S, Palmer G, Martin P, et al. IL-36 signaling amplifies Th1 responses by enhancing proliferation and Th1 polarization of naive CD4+ T cells. Blood. 2012;120(17):3478–3487. doi:10.1182/blood-2012-06-439026. 22968459

[82] Chen F, Qu M, Zhang F, et al. IL-36 s in the colorectal cancer: Is interleukin 36 good or bad for the development of colorectal cancer? BMC Cancer. 2020;20(1):92. doi:10.1186/s12885-020-6587-z. 32013927 PMC6998229

[83] Simoni Y, Fehlings M, Kløverpris HN, et al. Human innate lymphoid cell subsets possess tissue-type based heterogeneity in phenotype and frequency [published correction appears in Immunity. 2018;48(5):1060. Immunity. 2017;46(1):148–161. doi:10.1016/j.immuni.2016.11.005. 29768165

[84] Ikeda A, Ogino T, Kayama H, et al. Human NKp44+ group 3 innate lymphoid cells associate with tumor-associated tertiary lymphoid structures in colorectal cancer. Cancer Immunol Res. 2020;8(6):724–731. doi:10.1158/2326-6066.CIR-19-0775. 32229590

[85] Vilgelm AE, Richmond A. Chemokines modulate immune surveillance in tumorigenesis, metastasis, and response to immunotherapy. Front Immunol. 2019;10:333. doi:10.3389/fimmu.2019.00333. 30873179 PMC6400988

[86] Coppola D, Nebozhyn M, Khalil F, et al. Unique ectopic lymph node-like structures present in human primary colorectal carcinoma are identified by immune gene array profiling. Am J Pathol. 2011;179(1):37–45. doi:10.1016/j.ajpath.2011.03.007. 21703392 PMC3123872

[87] Tokunaga R, Nakagawa S, Sakamoto Y, et al. 12-Chemokine signature, a predictor of tumor recurrence in colorectal cancer. Int J Cancer. 2020;147(2):532–541. doi:10.1002/ijc.32982. 32191346 PMC9371443

[88] Väyrynen JP, Sajanti SA, Klintrup K, et al. Characteristics and significance of colorectal cancer associated lymphoid reaction. Int J Cancer. 2014;134(9):2126–2135. doi:10.1002/ijc.28533. 24154855

[89] Kim JH, Kim K-J, Bae JM, et al. Comparative validation of assessment criteria for Crohn-like lymphoid reaction in colorectal carcinoma. J Clin Pathol. 2015;68(1):22–28. doi:10.1136/jclinpath-2014-202603. 25322692

[90] Posch F, Silina K, Leibl S, et al. Maturation of tertiary lymphoid structures and recurrence of stage II and III colorectal cancer. Oncoimmunology. 2018;7(2):e1378844. doi:10.1080/2162402X.2017.1378844. 29416939 PMC5798199

[91] West NP, Dattani M, McShane P, et al. The proportion of tumour cells is an independent predictor for survival in colorectal cancer patients. Br J Cancer. 2010;102(10):1519–1523. doi:10.1038/sj.bjc.6605674. 20407439 PMC2869173

[92] Mesker WE, Junggeburt JM, Szuhai K, et al. The carcinoma-stromal ratio of colon carcinoma is an independent factor for survival compared to lymph node status and tumor stage. Cell Oncol. 2007;29(5):387–398. doi:10.1155/2007/175276. 17726261 PMC4617992

[93] Wang Q, Shen X, An R, et al. Peritumoral tertiary lymphoid structure and tumor stroma percentage predict the prognosis of patients with non-metastatic colorectal cancer. Front Immunol. 2022;13:962056. doi:10.3389/fimmu.2022.962056. 36189233 PMC9524924

[94] Salama P, Stewart C, Forrest C, et al. FOXP3+ cell density in lymphoid follicles from histologically normal mucosa is a strong prognostic factor in early-stage colon cancer. Cancer Immunol Immunother. 2012;61(8):1183–1190. doi:10.1007/s00262-011-1191-3. 22210551 PMC11029203

[95] Schweiger T, Berghoff AS, Glogner C, et al. Tumor-infiltrating lymphocyte subsets and tertiary lymphoid structures in pulmonary metastases from colorectal cancer. Clin Exp Metastasis. 2016;33(7):727–739. doi:10.1007/s10585-016-9813-y. 27449756 PMC5035322

[96] Zhang C, Wang XY, Zuo JL, et al. Localization and density of tertiary lymphoid structures associate with molecular subtype and clinical outcome in colorectal cancer liver metastases. J Immunother Cancer. 2023;11(2):e006425. doi:10.1136/jitc-2022-006425. 36759015 PMC9923349

[97] Weinstein AM, Chen L, Brzana EA, et al. Tbet and IL-36gamma cooperate in therapeutic DC-mediated promotion of ectopic lymphoid organogenesis in the tumor microenvironment. Oncoimmunology. 2017;6(6):e1322238. doi:10.1080/2162402X.2017.1322238. 28680760 PMC5486180

[98] Zhao H, Wang H, Zhou Q, et al. Insights into tertiary lymphoid structures in the solid tumor microenvironment: anti-tumor mechanism, functional regulation, and immunotherapeutic strategies. Cancer Biol Med. 2021;18(4):981–991. doi:10.20892/j.issn.2095-3941.2021.0029. 34553849 PMC8610165

[99] Tertiary Lymphoid Structures Validated as Biomarker. Cancer Discov. 2023;2023:OF1. doi:10.1158/2159-8290.CD-NB2023-0090. 37991371

[100] Meylan M, Petitprez F, Becht E, et al. Tertiary lymphoid structures generate and propagate anti-tumor antibody-producing plasma cells in renal cell cancer. Immunity. 2022;55(3):527–541.e5. doi:10.1016/j.immuni.2022.02.001. 35231421

[101] Mokhtari Z, Rezaei M, Sanei MH, et al. Tim3 and PD-1 as a therapeutic and prognostic targets in colorectal cancer: relationship with sidedness, clinicopathological parameters, and survival. Front Oncol. 2023;13:1069696. doi:10.3389/fonc.2023.1069696. 37035199 PMC10076872

[102] Saito A, Kitayama J, Horie H, et al. Metformin changes the immune microenvironment of colorectal cancer in patients with type 2 diabetes mellitus. Cancer Sci. 2020;111(11):4012–4020. doi:10.1111/cas.14615. 32794612 PMC7648042

[103] Zhao M, Yao S, Li Z, et al. The Crohn’s-like lymphoid reaction density: a new artificial intelligence quantified prognostic immune index in colon cancer. Cancer Immunol Immunother. 2022; May71(5):1221–1231. doi:10.1007/s00262-021-03079-z. 34642778 PMC10992499

[104] Li Z, Jiang Y, Li B, et al. Development and validation of a machine learning model for detection and classification of tertiary lymphoid structures in gastrointestinal cancers. JAMA Netw Open. 2023;6(1):e2252553. doi:10.1001/jamanetworkopen.2022.52553. 36692877 PMC10408275

